# Exploring the Sociodemographic Factors Influencing Women's Experiences With Domestic Violence and Their Help-Seeking Behaviors at the National Guard Health Hospitals

**DOI:** 10.7759/cureus.87337

**Published:** 2025-07-05

**Authors:** Awateif O Alsupiany, Hatem Aseeri, Hind A Ababtain, Jinan Z Shamou, Roaa M Abusulaiman, Salma O Alwahibah, Zubaida A Baashr, Fares F Al Harbi

**Affiliations:** 1 Department of Mental Health, King Abdulaziz Medical City, Riyadh, SAU; 2 King Abdullah International Medical Research Center, Ministry of National Guard Health Affairs, Riyadh, SAU; 3 Department of Medicine, King Abdulaziz Medical City, Riyadh, SAU; 4 Department of Mental Health, Prince Sultan Military Medical City, Riyadh, SAU; 5 College of Medicine, King Saud Bin Abdulaziz University for Health Sciences, Riyadh, SAU

**Keywords:** domestic abuse, partner violence, perpetrators, saudi women, sociodemographic data, women's mental health

## Abstract

Background

Domestic violence is recognized as a public health issue. It has adverse impacts on women's physical, mental, and reproductive well-being. This study aimed to ascertain the sociodemographic attributes of domestic violence in women who seek help at the National Guard Health (NGH) hospitals, as well as examine the psychological condition of the victims.

Methods

This was a retrospective-prospective, descriptive cross-sectional study conducted in four regions in Saudi Arabia (Riyadh, Jeddah, Al Madinah, and Al-Ahsa). The data collection tool was a questionnaire consisting mainly of three sections (the sociodemographic characteristics, abuse characteristics, and victim's knowledge and ways to get help), developed and validated by the study authors and field experts. Data were collected from the electronic data extraction system (BestCare) and through structured phone interviews conducted by a well-trained research team.

Results

A total of 292 women participated, with a median age of 31 years (IQR, 26-38). A percentage of 8.5% reported suffering violence from many people. Husbands were the foremost perpetrators (196, 67.1%), and physical violence represented 166 cases (57%). Among the victims, the prevalence of anxiety/spectrum disorder increased from 15 (5.2%) to 22 (7.5%); 59 (20.1%) reported suicide attempts, while 18 (6.1%) had suicidal ideation. Almost one-third (112, 33.3%) of the victims were referred to social service follow-up, 74 (22%) to the family therapy clinic, 33 (9.8%) to the psychiatry outpatient department, and 28 (8.3%) were admitted for protection. Violence was ongoing in 36 (12.3%) of the victims; hospitals and police were the primary sources of getting help (56, 18.4%). Filing a legal complaint and involving family members were cited as the most helpful way to help stop the violence, followed by health facility intervention as the next most beneficial modality by 34 (11.4%) of the victims, in which psychiatry was the most helpful. Victims know about the domestic violence team services mainly through official referrals from other teams, while the media has almost no role.

Conclusion

Our findings showed that domestic violence was prevalent, notably in the form of physical abuse. Most victims were the same age or younger than their perpetrators, and some had the same education level. Most of the perpetrators were military-employed husbands. The findings also reveal a mixed pattern in how victims of abuse sought help. Suicidal attempts and ideation were also commonplace among the victims. Hospitals and police were the primary sources of help for the victims. Although social media can play a crucial role in raising awareness and providing education, this aspect has often been overlooked.

## Introduction

Violence perpetrated against women is a significant issue in public health and an apparent infringement against the fundamental human rights of women [1]. Domestic violence refers to the act of using physical, psychological, or emotional aggression to injure or exert authority and control over another person. The offender is a member of the victim's "domestic environment," which includes an intimate partner, spouse, ex-intimate partner, family member, friend, or acquaintance [2]. In community studies, "domestic violence" is generally used to refer to partner abuse, particularly physical violence between a male and female partner, which is mainly perpetrated by the male partner [3]. However, "domestic violence" (or "family violence") can also be used to refer to abuse occurring in a domestic relationship (i.e., including abuse of children, elders, or siblings) [3]. In the literature, the terms "brawls," "intimate terrorists," "assault," and "domestic violence" are all suitable for arbitration tribunals for domestic violence. The authors of this study noticed that the most studied type of domestic violence was physical [4,5].

Globally, approximately one in three women has experienced physical and/or sexual violence at least once in their lives [6]. This issue is a worldwide concern, as indicated by reports stating that 25.4% of women in the European Region and 29.8% of women in the Americas endure physical or sexual abuse from their partners [1]. The prevalence rates in Southeast Asia and the Eastern Mediterranean regions are notably high, at 37.7% and 37%, respectively [1].

In Saudi Arabia, some studies conducted across different regions among women demonstrated that the lifetime prevalence of domestic violence in Riyadh was 43%, with 9% reporting physical violence and 22% reporting emotional violence [7]. Jeddah's lifetime prevalence was 33%, with a reported 34.8% physical violence and a reported 48.47% psychological violence [4], while in Al-Ahsa, lifetime domestic violence was 39.9%, with a reported 22.8% physical violence and a reported 29.1% mental violence like manipulation, isolation from family and friends, and intimidation [8] and 57.7% in Medina, with 25.7% of the abuse being physical and 96% of it being emotional [5]. Moreover, a meta-analysis was conducted to determine the prevalence and health outcomes of domestic violence among clinical populations in Arab countries. The study found that the estimated prevalence of lifetime exposure to any form of domestic violence was 73.3% [9].

Violence has adverse impacts on women's physical, mental, sexual, and reproductive well-being. It is associated with an increased risk of injuries, depression, anxiety disorders, unplanned pregnancies, and sexually transmitted infections, and it can potentially heighten the likelihood of contracting HIV in certain circumstances [10]. Furthermore, being subjected to domestic violence can cause significant distress and result in seeking medical attention at the emergency department. A limited number of studies have been conducted among emergency department patients in Ontario, one of which reveals that there were 10,935 visits to the emergency department related to domestic violence between 2012 and 2016. Out of the total number of visits, 8,878 (81.2%) were made by females, while 2,057 (18.8%) were made by males [11].

Consequently, domestic violence against women is linked to a range of detrimental implications, including the possibility of being killed. One study found that 13.5% of homicides were committed by an intimate partner [12]. Also, research indicates that 28% of domestic violence victims required hospitalization to receive medical treatment for their injuries, and 13% underwent significant surgical procedures [13]. Research indicates that women who have experienced abuse have a higher relative risk of developing mental disorders, specifically depression, anxiety/neuroses, and tobacco use, at 3.26, 2.73, and 2.31, respectively, compared to women who have not experienced abuse [14]. In addition, the victims have a twofold increased likelihood of making multiple suicide attempts [15].

In terms of healthcare, civilian and military hospitals serve distinct populations by offering different services and operating under varied organizational structures. A 2017 survey highlighted that a substantial majority of physicians (78.3%) recognized the pivotal role of social services in managing violence cases. Moreover, 64.4% of victims appreciated the ongoing support that facilitated communication with the medical team at King Abdul Aziz Military Hospital [16].

There is a deficit of research examining domestic violence within Saudi communities. The majority of these studies have focused on screening for domestic violence in a primary care setting. To the best of our understanding, there have been no studies conducted in Saudi Arabia that examine domestic violence among women who seek assistance and intervention at hospitals. For this reason, the objective of this study is to ascertain the sociodemographic attributes of domestic violence in women who seek help at the National Guard Health (NGH) hospitals, as well as examine the psychological condition of the victims. This research can provide a foundation for developing optimal management strategies and providing high-level care.

## Materials and methods

Design and sample

This is a retrospective-prospective descriptive cross-sectional study that was conducted at the NGH hospitals across Saudi Arabia from January 2016 to December 2020 among women aged 18 and above who experienced or have a history of domestic violence or abuse and who sought help in the hospital.

Data were withdrawn from all general hospitals and primary care centers, including different departments such as the medical, surgical, pediatric, antenatal and postpartum, rehabilitation and long-term care wards, intensive care units, a cardiac center, an emergency and trauma department, obstetrics and gynecology services, outpatient clinics, and dental services across four sites: Riyadh, Jeddah, Al Madinah, and Al-Ahsa, Saudi Arabia. The total sample of the study consisted of 361 participants; out of these, 33 (9.14%) refused to participate, 36 (9.97%) were unreachable, and 292 (80.89%) agreed to participate and were enrolled in the study, resulting in an 81% response rate.

Data collection and procedures

Data were collected using an electronic data extraction system (BestCare) from patient files, through questionnaires, and through structured interviews to collect missing data. These data collection activities were carried out by a research team trained in the data collection process prior to the data collection stage.

The questionnaire was developed by the research team and validated by experts in the field. To ensure content validity, the initial draft of the questionnaire was reviewed by a panel of experts in the fields of psychiatry and psychology. These experts evaluated the questionnaire for relevance, clarity, and comprehensiveness in measuring the intended constructs.

Based on the feedback from the expert panel, revisions were made to improve question phrasing, eliminate ambiguities, and ensure cultural and contextual appropriateness. A pilot test was then conducted with a small sample (n = 10) from the target population to assess the questionnaire's clarity, internal consistency, and completion time. Minor adjustments were made based on the findings of the pilot test. The questionnaire is composed of three sections. The first section covered respondents' sociodemographic characteristics, including age, level of education, income, and occupation. The second section was about characteristics of abuse, general health, and mental health. The third section assessed the victim's knowledge and resources for seeking help. In addition, the questionnaire included questions about the risks and protective factors of intimate partner violence in the four domains of domestic abuse against women: physical, psychological, sexual, and economic abuse. The psychological condition of participants was assessed through a set of researcher-developed items aimed at identifying symptoms such as persistent sadness, sleep disturbances, anxiety, and emotional distress. While not based on standardized diagnostic tools, this assessment was provided by mental health professionals. As part of the standard clinical protocol at the NGH hospitals, all participants who presented with concerns related to domestic violence underwent a psychiatric evaluation conducted by qualified mental health professionals. These assessments were conducted in relevant clinical settings, including emergency departments, outpatient clinics, and inpatient units, by psychiatrists and clinical psychologists trained in diagnostic evaluation.

The candidates who are still exposed to domestic violence were offered online counseling sessions by a family therapist who is working at the NGH, as well as physical appointments with the psychologist or psychiatrists if needed.

Statistical analysis

Statistical analysis was done using IBM SPSS Statistics for Windows, Version 25 (Released 2017; IBM Corp., Armonk, New York). Frequency and percentages were used for the categorical data, while means ± standard deviation (SD) or median (interquartile range), as appropriate, were used for continuous data to describe the sociodemographic statistics of domestic violence victims, demographic and behavioral characteristics of perpetrators, psychiatric diagnosis and service intervention for victims, and victims' knowledge and ways to get help.

## Results

**Figure 1:**
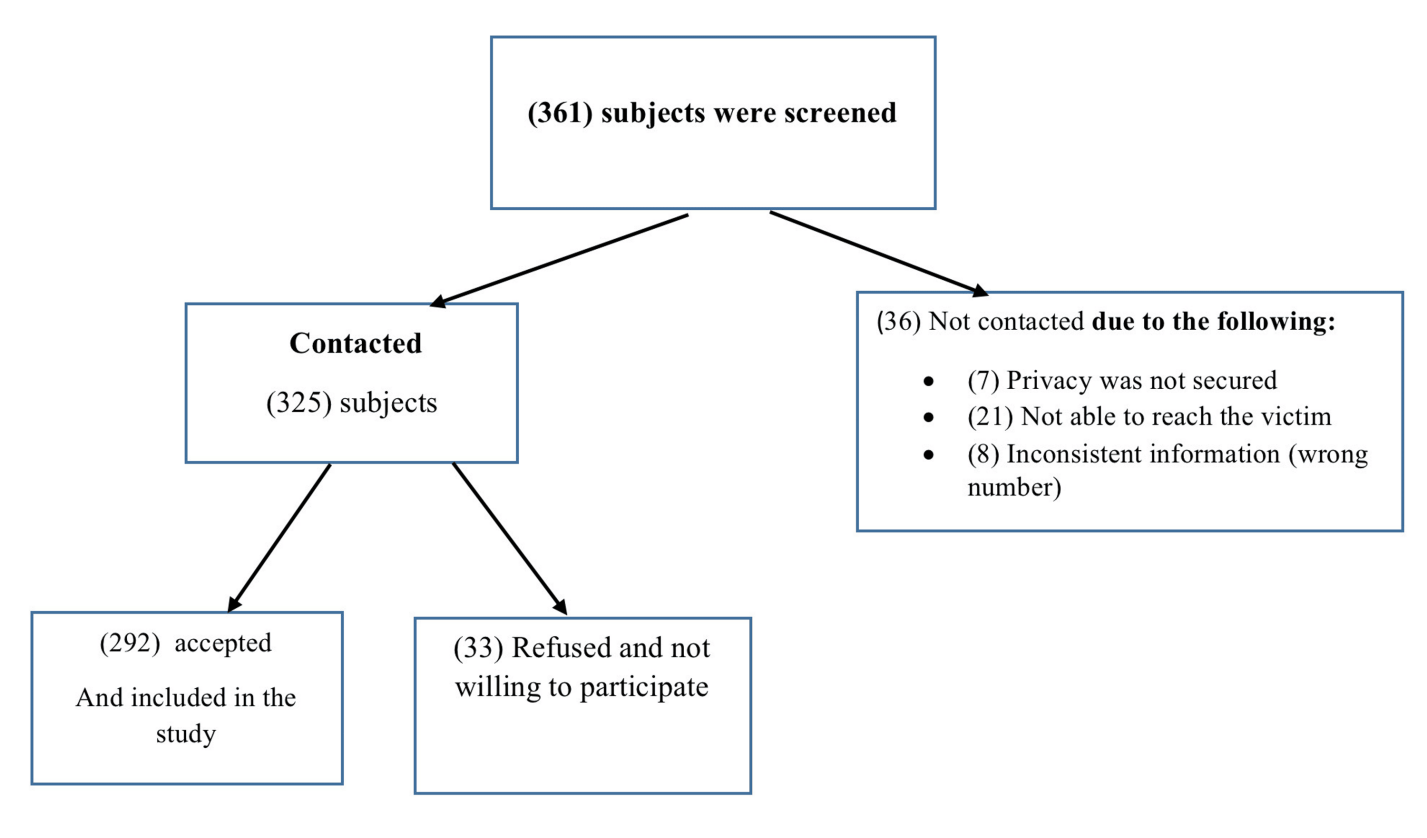
Flow diagram for the study participants Flowchart created by the authors.

A total of 292 women (80.89%) participated in this study. The median age of the participants was 31 years (IQR 26-38); most of them were married (209, 71.5%), 204 (69.4%) had children, and the number of children ranged from one to three, with the highest percentage (109, 37.3%) having two children. Almost half (135, 46.2%) of the participants were unemployed, while the highest percentage (92, 31.5%) had a high school education. According to the available data on the type of residence, 59 (20.2%) lived in their own villa, and nearly half (145, 49.6%) were financially independent. Half (146, 50%) of the participating victims reported that their original family is their primary source of support. Six individuals (2.1%) reported having disabilities, and 8.5% suffered violence from multiple people. The data are presented in Table 1 and Figure 1.

**Table 1:** Sociodemographic characteristics of domestic violence victims

Parameter		Frequency (N=292)	Percentage
Age (median, IQR)	31 (26–38)	-	-
Gender	Female	292	100
Marital status	Married	209	71.5
	Divorced	25	8.5
	Single	50	17.1
	Widowed	3	1.1
	Not mentioned/documented	5	1.7
Years of marriage	≤ 5 years	115	39.4
	6–20	72	24.6
	≥ 21	18	6.2
	Not mentioned/documented	37	12.7
Presence of kids	Yes	204	69.4
	No	39	13.3
	Not documented	2	0.7
	Not applicable	5	1
Number of children	None	39	13.3
	1–3 children	109	37.3
	4–6 children	57	19.5
	≥ 7	32	10.9
	Not applicable	50	17.1
	Not documented	5	1.7
Employment status	Student	33	11.2
	Unemployed (stay home)	135	46.2
	Governmental employee	53	18
	Private sector employee	34	11.6
	Others	15	5.1
	Not documented	22	7.5
Education level	Illiterate	13	4.4
	Primary/intermediate	72	24.6
	High school	92	31.5
	Diploma	35	11.9
	College/postgraduate	80	27.4
Type of residence	Rented apartment	53	18
	Rented villa	8	2.7
	Owned apartment	22	7.5
	Owned villa	59	20.2
	Other	7	2.4
	Not mentioned/documented	143	48.9
Financial dependency	Yes	112	38.4
	No	145	49.6
	Not mentioned	35	11.9
Type of support	No support	3	1
	Original family	146	50
	Extended family	4	1.4
	Friendships	3	1
	Other	10	3.4
	Not applicable	50	17.1
	Not documented	76	26
Does the victim suffer from any kind of disability?	Yes	6	2.1
	No	224	76.7
	Not documented	62	21.2
Does the victim suffer violence from multiple people?	Yes	25	8.5
	No	265	90.7
	Not documented	2	0.7

Table 2 presents the characteristics of the perpetrators, victims, and healthcare services. Husbands represented almost two-thirds of the perpetrators (196, 67.1%), followed by brothers (49, 16.8%). The mean age of the perpetrators was 40.5±11.1 years; most of them were not drug abusers (207, 70.4%), 35 (11.9%) were smokers, 30 (10.2%) were taking recreational drugs, and eight (2.7%) were drug abusers (combined). Regarding the type of violence, 166 (56%) was physical violence, 49 (16.8%) was combined (physical, sexual, and emotional), and 46 (15.7%) was combined physical and verbal. According to the available data on educational level, the highest percentage of perpetrators (92, 31.5%) had a high school academic level, and 116 (39.5%) were employees.

**Table 2:** Demographic and behavioral characteristics of perpetrators of violence

Parameter		Frequency (N=292)	Percentage
Perpetrator	Husband	196	67.1
	Brother	49	16.8
	Sister	2	0.7
	Father	16	5.5
	Mother	4	1.4
	Multiple people	25	8.5
	Not documented	2	0.7
Age of perpetrator	(Mean ± SD)	40.5 ± 11.1	
	Documented	131	44.8
	Not documented	161	55.2
Drug abuse of the perpetrator	None	207	70.4
	Smoking	35	11.9
	Recreational drugs	30	10.2
	Alcohol	5	1.7
	Amphetamine	2	0.7
	Not documented	7	2.4
	Combined	8	2.7
Type of violence	Physical	166	56.8
	Emotional	1	0.3
	Sexual	1	0.3
	Verbal	5	1.7
	Physical, verbal	46	15.7
	Combined (physical, sexual, emotional)	21	7.2
	Other	49	16.8
	Not documented	3	1.1
Education level of the preparator	Illiterate	4	1.4
	Primary/intermediate	23	7.8
	High school	92	31.5
	Diploma	21	7.2
	College/postgraduate	20	6.8
	Not documented	132	45.2
Employment status of the perpetrator	Unemployed	36	12.2
	Employed	116	39.5
	Retired	10	3.4
	Not mentioned	126	42.8
	Not documented	6	2.1

For the previous psychiatric diagnoses of the victims, 236 (80.8%) were free, while 27 (9.2%) had mood/affective disorder, and 15 (5.2%) had anxiety/spectrum disorder. At presentation, 7.5% of the victims were diagnosed as having anxiety/spectrum disorder, and the same percentage (22, 7.5%) as having mood/affective disorders. Suicide attempts were reported from 59 (20.1%) of the victims, while 18 (6.1%) had suicide ideation. The vast majority (284, 97.2%) of the victims were non-drug abusers; six (2%) of them were smokers, while two (0.7%) were taking recreational drugs. For the provided healthcare services, one-third (112, 33.3%) of the victims were referred to social service follow-up, 74 (22%) to the family therapy clinic, 33 (9.8%) to the psychiatry outpatient department (OPD) follow-up, and 28 (8.3%) were admitted for protection. For psychotropic medication use, 42 (14%) were using antidepressants, and eight (2.7%) were using antipsychotic drugs. For the medical history, 212 (64.4%) of the victims have no medical history, 30 (9.2%) have endocrine disorders, and 21 (6.4%) have cardiac diseases (Table 3).

**Table 3:** Psychiatric diagnosis and service interventions for the victims OPD: outpatient department; GI: gastrointestinal; DLP: dyslipidemia

Parameter		Frequency (N=292)	Percentage
Previous psychiatric diagnosis of the victim	None	236	80.8
	Anxiety spectrum disorder	15	5.2
	Mood/affective disorder	27	9.2
	Intellectual disability	1	0.3
	Schizophrenia	1	0.3
	Not mentioned	12	4.1
Diagnosis at presentation of victims	None	232	79.5
	Anxiety spectrum disorder	22	7.5
	Mood/affective disorder	22	7.5
	Cluster B personality disorder	2	0.7
	Intellectual disability	1	0.3
	Schizophrenia	1	0.3
	Not mentioned	12	4.1
Suicide assessment of the victim	None	214	73.3
	Ideation	18	6.1
	Attempt	59	20.1
	Death wishes	1	0.3
Drug abuse of the victim	None	284	97.2
	Smoking	6	2
	Recreational drugs	2	0.7
Type of service provided (multiple answers)	Admission for protection	28	8.3
	Psychiatry OPD follow-up	33	9.8
	Psychology OPD follow-up	18	5.3
	Social service follow-up	112	33.3
	Family therapy clinic	74	22
	No intervention	36	10.7
	Medication	33	9.8
	Not documented	2	0.6
Psychotropic medication (multiple answers)	None	242	80.9
	Antidepressant	42	14
	Antipsychotic	8	2.7
	Benzodiazepine	5	1.7
	Not documented	2	0.7
Medical history (multiple answers)	None	212	64.4
	Not mentioned	14	4.3
	GI disorder	6	1.8
	Cardiac disease	21	6.4
	Endocrine disorder	30	9.2
	Neurological disorder	3	0.9
	DLP	16	4.9
	Others	27	8.2

As shown in Table 3, the violence was ongoing in 36 (12.3%) of the victims. Based on the documented data, 117 (40%) know where to go. Hospitals and police were the sources of help for 56 (18.4%) and 52 (17.1%) of the victims, respectively, while the family was the source for 34 (11.1%). Filing a legal complaint and involving family members were cited as the most helpful way to help stop the violence, followed by health facility intervention as the next most beneficial modality by 34 (11.4%) of the victims, in which psychiatry was the most helpful. Victims primarily learn about the domestic violence team services in NGH through official referrals from other teams, whereas the media plays a minimal role (Table 4).

**Table 4:** Victims' knowledge and ways to get help

Parameter		Frequency (N=292)	Percentage
Is the violence still ongoing?	Yes	36	12.3
	No	112	38.4
	Not documented	144	49.3
Do you know where to go for help?	Yes	117	40.1
	No	30	10.3
	Not documented	145	49.6
Where do you get help from? (multiple answers)	None	153	50.2
	Police	52	17.1
	Hospital	56	18.4
	Family	34	11.1
	Friends	3	0.9
	Other	7	2.3
What was the most helpful way to stop the violence? (multiple answers)	None	152	51.2
	Filing a legal complaint	56	18.8
	Involved family members	39	13
	Health facility intervention	34	11.4
	Other	16	5.4
Referrals made by the NGH domestic violence response team (multiple answers)	None	174	59.2
	Psychiatry	27	9.2
	Psychology	11	3.7
	Family therapy	26	8.8
	Family dispute clinic	13	4.4
	Other	43	14.6
From where the victim heard about the domestic violence team services in NGH (multiple answers)	Not heard about it	34	10.9
	Media	5	1.6
	Advice from another physician	30	9.6
	Official referrals from other	88	28.2
	Team services	28	9
	Friends/family	127	40.7

## Discussion

In this cross-sectional study, we aimed to identify the sociodemographic profile of victims of domestic abuse among women seeking care at the NGH, as well as to analyze the psychological characteristics of these victims at presentation. Our findings revealed that husbands perpetrated 67.1% of the violence, which is consistent with a local study that showed the perpetrator was the husband in 76.9% of cases, and the husband's family was the perpetrator in 3.8% of cases [17]. A review of previous local studies, some conducted in healthcare settings and others through community-based surveys, found that the prevalence of intimate partner violence in Saudi Arabia ranges more broadly from 20% to 39% [18]. This study does not reflect the prevalence of domestic violence against women but rather reflects the sociodemographic characteristics of the victims.

In the current study, all victims were predominantly of a similar or younger age than their perpetrators. This demographic trend aligns with findings from a 2017 study at a family medicine center in Riyadh, which highlighted the vulnerability of young women to such incidents [19]. Furthermore, according to the WHO, younger women are generally more susceptible to intimate partner violence [20]. These consistent findings across multiple studies suggest that the age dynamics between victims and perpetrators, with victims being the same age as or younger than the perpetrators, is an essential factor to consider in understanding the dynamics and characteristics of the explored incidents.

This study further reveals that the victims typically have no more than three children, echoing previous observations of smaller family units and insufficient family income contributing to violence [19]. Educational attainment among the women in this study was found to be on par with that of the perpetrators, contrasting with earlier research that associated higher education levels with women experiencing abuse [19,20]. A systematic review of literature on domestic violence in Saudi Arabia from 2009 to 2017 identified the educational levels of both victims and their spouses as a common risk factor, with victims often possessing lower educational qualifications [21]. While educational status is not a definitive indicator of economic stability, it is noteworthy that approximately half of the victims in this study were unemployed, a proportion exceeding that of the perpetrators, suggesting a potential link to financial dependency.

Occupationally, perpetrators were frequently employed in military roles [19,20], and national data indicate a significant correlation between domestic violence and substance abuse by the spouse [21]. Consistent with a prior study, this research also found a higher incidence of smoking among perpetrators [19], reinforcing the need to consider these behavioral patterns when addressing domestic violence.

The findings of this research underscore the potential for utilizing these insights to refine management strategies and enhance the quality of care for victims of violence, with a particular emphasis on mental health outcomes. Our research establishes a notable incidence of mental health issues linked to violence, with an elevated risk observed for conditions such as suicidality and mood and anxiety disorders. These findings were particularly evident at the initial evaluation of the victims, who had no previous record of these mental health symptoms in their medical history. A scoping review conducted in 2023 synthesized evidence from studies between 2010 and 2020, revealing that intimate partner violence can lead to mental health problems like depression and post-traumatic stress disorder (PTSD) [22].

Additionally, this study observed that victims of violence also suffer from endocrine disorders (9.2%) and cardiac diseases (6.4%). These health consequences align with previous findings [8,19,23,24] that indicate a range of impacts, including chronic pain, poor overall health, somatic complaints, suicidal thoughts, and potential substance abuse [8,23].

The findings reveal a mixed pattern in how victims of abuse sought help. Nearly half reported knowing where to turn; some were more likely to go to the hospital, while others were more likely to go to the police. However, the most helpful ways to stop the violence were cited as filing legal complaints and involving family members. Interestingly, an equal percentage of victims found family and health facilities to be the most supportive sources, underscoring the importance of both family awareness and well-trained medical professionals, as recommended by a 2014 local study [25]. While some individuals confided in their doctors about the violence, relatively few reported it to the police or social services due to fears of disrupting their marital relationship, losing their children, concerns about bringing shame to the family, facing family rejection, or getting divorced [26]. In fact, in other regions in Saudi Arabia, most survivors at military hospitals do not seek help. Therefore, it is necessary to encourage help-seeking behavior in Saudi Arabia and raise understanding of one's legal rights [26].

Our results show that the most common type of violence reported was physical abuse, affecting 56.8% of women at the NGHs across four regions. This prevalence is notably higher than the worldwide average of 30% or the Eastern Mediterranean regional rate of 37% [20]. This study is among the first in Saudi Arabia to address physical violence, specifically within the military sector. In contrast, previous Saudi studies have primarily assessed physical abuse in clinical settings such as primary healthcare centers. The higher rate observed in the current military hospital-based study underscores the importance of expanding research to understand the unique dynamics and patterns of abuse within this under-explored sector. The discrepancies in the prevalence of domestic abuse found in various Saudi studies can be attributed to multiple methodological factors, such as differences in study design, duration, setting, and data collection methods (survey-based vs. interview). Notably, studies with smaller sample sizes tended to report higher prevalence rates of physical violence. For instance, a study in Arar among 208 participants reported an 80.7% prevalence of physical violence [17], and a survey in Jeddah with 200 participants found a 44.5% prevalence of physical violence [23]. In comparison, our study, which had 292 participants, found a 56.8% prevalence of physical violence.

Another factor contributing to the variation in prevalence rates is the sociodemographic profile of the participants. Women from different cities may have diverse cultural backgrounds influencing their experiences of violence. It is worth noting that this study employed a retrospective design, in which data were initially extracted from existing medical records. To enhance data completeness, a prospective follow-up was attempted through structured telephone interviews with participants to collect any missing information. However, not all participants were reachable during the follow-up period due to factors such as invalid contact information, lack of response, or refusal to continue participation. As a result, some variables still contain missing values. However, our study is unique in that it was conducted prospectively and cross-sectionally at King Abdul Aziz Medical City across four sites: Riyadh, Jeddah, Al Madinah, and Al-Ahsa. This multi-site approach may explain the higher rate of reported cases, as it reflects greater accessibility and potential cultural factors, such as fear of the partner's reaction and concerns about facing disappointment from military health sectors. These aspects highlight the complex interplay of social and cultural issues, including the social taboo surrounding domestic violence and the desire to avoid social complications.

This study aims to enhance awareness and provide a foundation for developing effective management strategies and delivering high-quality care. Studies underscored several consequences for forthcoming practice, policy, and research. It was advised that specialists in the domain of domestic violence should remain current, adequately trained, and formally qualified to address issues related to domestic abuse. This group comprises medical personnel and legal professionals [22,24].

Limitations of the study

One limitation of the study was the reluctance of many victims to respond to or answer the survey due to their ongoing situation of living with the abuser. This non-response bias could have affected the sample's representativeness and potentially skewed the findings. Another challenge in the study was that the registered contact number used to identify participants was the victim's number. This identification method may have compromised the confidentiality and anonymity of the participants, potentially influencing their willingness to participate or provide accurate information. Also, some participants refused to answer specific questions or provide detailed information about their experiences because they did not want to remember or revisit the traumatic incidents they had endured. This limitation could have affected the completeness and accuracy of the data collected. Another limitation of the study was the fear of social stigma associated with being identified as a victim of abuse. This fear might have discouraged some individuals from participating or providing truthful responses, potentially leading to underrepresentation or biased results. Considering these limitations when interpreting the study's findings and applying them to a broader population or context is important.

## Conclusions

To sum up, our findings showed that domestic violence was prevalent, notably in the form of physical abuse. All victims were predominantly of a similar or younger age and were on the same level of education as their perpetrators. Perpetrators were mostly husbands who held military roles. Suicidal attempts and ideation were also commonplace among the victims. The findings also reveal a mixed pattern in how victims of abuse sought help, some going to hospitals and others to the police. However, the most helpful ways to stop the violence were cited as filing a legal complaint and involving family members.

To further expand our understanding of the topic at hand, it is crucial to recommend a future study that delves deeper into the complexities and nuances of the sociodemographic profile of victims of domestic abuse among women seeking care, as well as the psychological character of the victims at presentation. By conducting additional research, we can uncover new insights, challenge existing theories, and ultimately contribute to advancing knowledge and practice in this area. It would benefit academia and have practical implications for real-world applications. We strongly urge the conduct of a future study locally, particularly among military hospitals, to build upon our current understanding and make meaningful contributions.

## Appendices

**Table 5:** Original questionnaire

Part One: Sociodemographic Characteristics of Domestic Violence Victims	
Gender	Female
Age	Specify
Marital status	Married
	Divorced
	Single
	Widow
	Not mentioned/documented
Years of marriage	≤ 5 years
	6–20
	≥ 21
	Not documented/not mentioned
Presence of kids	Yes
	No
	Not documented
	Not applicable
Number of children	None
	1–3 children
	4–6 children
	≥ 7
	Not applicable
	Not documented
Employment status	Student
	Unemployed (stay home)
	Governmental employee
	Private sector employee
	Others
	Not documented
Educational level	Illiterate
	Primary/intermediate
	High school
	Diploma
	College/postgraduate
Mode of living	Rented apartment
	Rented villa
	Owned apartment
	Owned villa
	Other
	Not mentioned/documented
Financial dependency	Yes
	No
	Not mentioned
Type of support	No support
	Original family
	Extended family
	Friendships
	Other
	Not applicable
	Not documented
Does the victim suffer from any kind of disability?	Yes
	No
	Not documented
Does the victim suffer violence from multiple people?	Yes
	No
	Not documented
Part Two: Demographic and Behavioral Characteristics of Perpetrators of Violence	
Perpetrator	Husband
	Brother
	Sister
	Father
	Mother
	Multiple people
	Other
	Not documented
	Documented
Age of perpetrator	Specify
	Not documented
	Not mentioned
Drug abuse of the perpetrator	None
	Smoking
	Recreational drugs
	Alcohol
	Amphetamine
	Not documented
	Combined
Type of violence	Physical
	Emotional
	Sexual
	Verbal
	Physical, verbal
	Combined (physical, sexual, emotional)
	Other
	Not documented
Educational level of the perpetrator	Illiterate
	Primary/intermediate
	High school
	Diploma
	College/postgraduate
	Not documented
Employment status of the perpetrator	Unemployed
	Employed
	Retired
	Not mentioned
	Not documented
Part Three: Psychiatric Diagnosis and Service Interventions for the Victims	
Previous psychiatric diagnosis of the victim	No
	Yes
	Specify, if yes
	Not mentioned
Diagnosis at presentation of the victim	No
	Yes
	Specify
	Not mentioned
Suicide assessment of the victim	None
	Ideation
	Attempt
	Death wishes
	Not mentioned
Drug abuse of the victim	None
	Smoking
	Recreational drugs
	Not mentioned
Type of service provided (You can choose multiple answers)	Admission for protection
	Psychiatry OPD follow-up
	Psychology OPD follow-up
	Social service follow-up
	Family Therapy clinic
	No intervention
	Medication
	Not documented
Psychotropic medication (You can choose multiple answers)	None
	Antidepressant
	Antipsychotic
	Benzodiazepine
	Not documented
Medical history (You can choose multiple answers)	None
	Not mentioned
	Specify
	Others
Part Four: Victim’s Knowledge and Ways to Get Help	
Is the violence still ongoing?	Yes
	No
	Not documented
Do you know where to go for help?	Yes
	No
	Not documented
Where do you get help from? (multiple answers)	None
	Police
	Hospital
	Family
	Friends
	Other
What was the most helpful way to stop the violence? (multiple answers)	None
	Failing legal complaint
	Involved family members
	Health facility intervention
	Other
Health facility intervention (multiple answers)	None
	Psychiatry
	Psychology
	Family therapy
	Family dispute clinic
	Other
From where did the victim hear about the domestic violence team services in NGH (multiple answers)	Not heard about it
	Media
	Advice from another physician
	Official referrals from other
	Team services
	Friends/family
